# Predictive Value of Interim PET/CT in DLBCL Treated with R-CHOP: Meta-Analysis

**DOI:** 10.1155/2015/648572

**Published:** 2015-02-01

**Authors:** Na Sun, Jinhua Zhao, Wenli Qiao, Taisong Wang

**Affiliations:** Department of Nuclear Medicine, Shanghai First People's Hospital, Shanghai Jiao Tong University, Shanghai 200080, China

## Abstract

*Objective.* We conducted a meta-analysis to evaluate the predictive value of interim ^18^F-FDG PET/CT in patients with DLBCL treated with R-CHOP chemotherapy.* Methods.* We searched for articles published in PubMed, ScienceDirect, Wiley, Scopus, and Ovid database from inception to March 2014. Articles related to interim PET/CT in patients with DLBCL treated with R-CHOP chemotherapy were selected. PFS with or without OS was chosen as the endpoint to evaluate the prognostic significance of interim PET/CT.* Results.* Six studies with a total of 605 cases were included. The sensitivity of interim PET/CT ranged from 21.2% to 89.7%, and the pooled sensitivity was 52.4%. The specificity of interim PET/CT ranged from 37.4% to 90.7%, and the pooled specificity was 67.8%. The pooled positive likelihood ratio and negative likelihood ratio were 1.780 and 0.706, respectively. The explained AUC was 0.6978 and the *Q* ^*^ was 0.6519.* Conclusions.* The sensitivity and specificity of interim PET/CT in predicting the outcome of DLBCL patients treated with R-CHOP chemotherapy were not satisfactory (52.4% and 67.8%, resp.). To improve this, some more work should be done to unify the response criteria and some more research to assess the prognostic value of interim PET/CT with semiquantitative analysis.

## 1. Introduction

The use of positron emission tomography/computed tomography (PET/CT) with ^18^F-fluoro-2-deoxy-D-glucose (^18^F-FDG) for staging, monitoring treatment, and restaging in patients with lymphoma has remarkably expanded in recent years. Numerous studies reported that patients with a negative scan showed both a better progression-free (PFS) and a better overall survival (OS) and these results helped clinicians make further treatment decisions. Diffuse large B cell lymphoma (DLBCL) is the major histologic subtype of aggressive non-Hodgkin lymphoma (NHL) and comprises about 30% of it [1]. Most patients with DLBCL can be cured by chemotherapy, with or without radiotherapy. The most widely adopted first-line therapy is R-CHOP (rituximab, cyclophosphamide, doxorubicin, vincristine, and prednisolone). However, still about 20–40% of patients cannot be cured with R-CHOP and may need salvage therapy, such as high-dose chemotherapy followed by autologous stem-cell transplant (ASCT) [2]. Thus, it is important to identify the poor responders to first-line treatment, in order to switch them to alternative treatments as early as possible. The ^18^F-FDG PET/CT scan is recommended at the baseline and end of treatment for DLBCL patients [3]. The interim ^18^F-FDG PET has shown high predictive value in Hodgkin's lymphoma (HD). However, the role of interim PET/CT in patients with NHL, including DLBCL, is still unconfirmed [4, 5]. The variability in patient population, treatment regimens, timing of interim PET, and nonstandardized FDG-PET interpretation criteria evident in these studies were the reasons to state that no reliable conclusions could be drawn from their analysis of interim PET used in DLBCL [6, 7].

In this paper, we performed a meta-analysis concentrating on the interim ^18^F-FDG PET/CT in DLBCL patients treated with R-CHOP chemotherapy.

## 2. Methods

### 2.1. Literature Search

**Figure 1:**
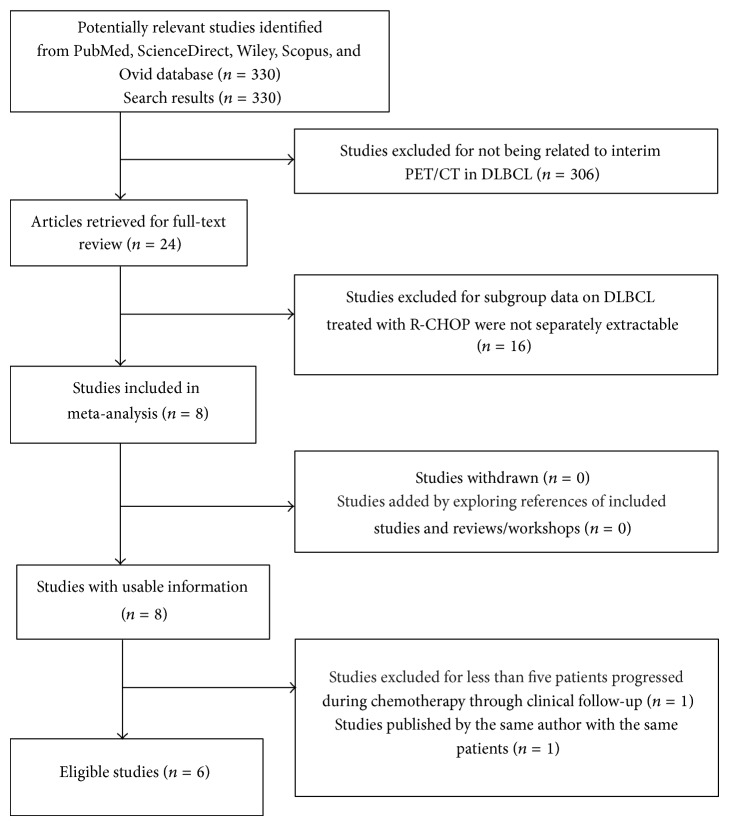
Flow diagram of study selection process for meta-analysis.

We conducted a search on the PubMed, ScienceDirect, Wiley, Scopus, and Ovid database for articles published in English from inception to March 2014, using keywords (PET or positron emission tomography), (DLBCL or diffuse large B cell lymphoma), humans, and English. Full-text articles were reviewed when abstracts did not provide sufficient information for determination. Furthermore, the reference lists of retrieved articles were examined for additional relevant studies. Exact search strategies can be found in Figure 1.

### 2.2. Study Selection

Two investigators independently reviewed the abstracts and further examined the full-text articles to select studies that met the inclusion criteria as follows: (1) studies that evaluated the predictive value of PET/CT performed between the first and the fourth cycle of first-line chemotherapy (R-CHOP) for patients with DLBCL; (2) studies that evaluated at least 10 patients and included at least five patients who progressed during chemotherapy through clinical follow-up; (3) studies using positive and negative results of ^18^F-FDG PET/CT as a predicting factor according to SUV_max_ value or visual analysis.

Besides, when a study included patients who were treated with R-CHOP and other chemotherapy, we included it only if subgroup data on R-CHOP were separately extractable. We excluded abstracts, editorials, comments, letters, review articles, and studies that enrolled patients with HIV associated or posttransplant lymphoproliferative disorders.

Many studies did not meet all the inclusion criteria but did partially include a relevant patient population. For these studies, we contacted the authors for relevant individual patient or subgroup data. When there was no response after 4 weeks, another correspondence was sent. When there was no response after the third communication attempt, we considered the request rejected.

### 2.3. Data Extraction and Quality Assessment

Two investigators independently reviewed the selected studies and retrieved data on author, publication year, patient characteristics, and study design. PFS with or without OS data was chosen as the endpoint to evaluate the prognostic significance of interim PET/CT. For each study, the numbers of true-positive (TP), false-positive (FP), false-negative (FN), and true-negative (TN) results were calculated. Study quality was assessed with QUADAS (quality assessment of studies of diagnostic accuracy included in systematic reviews) checklist and with maximum score, 14 [13].

### 2.4. Data Analysis

We constructed a 2 × 2 contingency table consisting of TP, FP, FN, and TN results. We calculated sensitivity and specificity for each study. The summary sensitivity, specificity, and positive and negative likelihood ratios (LRs) of the included studies were also calculated. We assessed study heterogeneity by plotting sensitivity and specificity in the receiver operating characteristic (ROC) space and drew summary ROC curves and confidence regions for summary sensitivity and specificity. As a global measure for the summary ROC curves, we estimated the *Q* ^*^ statistic, the point on the ROC curve where sensitivity and specificity were equal. Data analyses were conducted with Meta-Disc1.4 software. All tests were two-sided and statistical significance was defined as a *P*  value < 0.05.

## 3. Results

### 3.1. Eligible Studies

We identified 330 potentially relevant studies, of which 324 studies were rejected. The detailed study selection process was described in Figure 1. 306 studies were excluded for not being related to interim PET/CT in DLBCL. 16 studies were excluded because subgroup data on DLBCL treated with R-CHOP were not separately extractable. 1 study was excluded because less than 5 patients progressed during chemotherapy through clinical follow-up, and 1 study was excluded for being a duplicate study. Finally, there were six studies included in the final analysis.

The baseline characteristics of the six included studies were shown in Table 1. A total of 605 DLBCL patients between March 2004 and December 2010 were included in this meta-analysis. The age of patients ranged from 16 to 81. Interim PET/CT was performed after 2–4 cycles of first-line chemotherapy (R-CHOP) for patients with DLBCL. PFS with or without OS was chosen as the endpoint to evaluate the prognostic significance of interim PET/CT. The follow-up period ranged from 12 to 81 months. Because of the absence of consensus on criteria, the ^18^F-FDG PET/CT scans were designated positive or negative according to visual and semiquantitative methods. Visual interpretation used the International Harmonization Project (IHP) criteria and Deauville five-point scale (5-PS) criteria when semiquantitative method used the parameter of ΔSUV_max⁡_ (ΔSUV_max⁡_ = (%)100 × [SUV_max⁡_(initial) − SUV_max⁡_(interim)]/SUV_max⁡_(initial)), with different optimal cutoff values [14, 15]. The detailed criteria of the 6 included studies were as follows: three studies used the visual method while the other three used both visual and semiquantitative method (ΔSUV_max_). Among the three studies used both methods, the subgroup data of two studies [2, 8] were extracted separately as follows: (a) the subgroup data of the visual method; (b) the subgroup data of the semiquantitative method. Three of these studies achieved definite statistical significance while the other three showed undetermined results.

### 3.2. Quality Assessment

Two reviewers independently assessed the quality items and discrepancies were resolved by discussion. The global quality score ranged from 11 to 13 (Table 1).

### 3.3. Data Analysis

**Figure 2:**
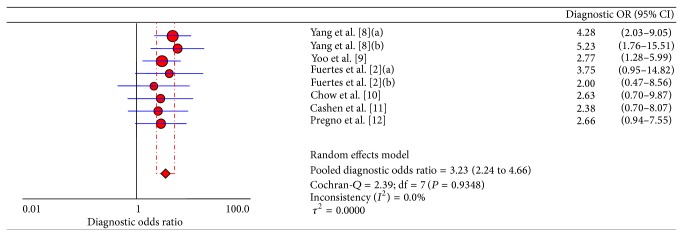
Diagnostic OR of 6 included studies for assessing the prognostic value of interim PET/CT. Two studies [2, 8] used both visual and semiquantitative methods (ΔSUV_max⁡_) and the data of both methods was separately extractable. (a) is the visual group and (b) is the semiquantitative group.

Heterogeneity is a potential problem when interpreting the results of meta-analysis. The threshold effect must be considered firstly in test accuracy studies, which arises when differences exist in sensitivity and specificity due to different cut-off or threshold used in different studies to define a positive or negative test result [16, 17]. We used Spearman correlation coefficient to analyze the threshold effect, and its value was 0.970 (*P* = 0.000), which indicated that there was heterogeneity from threshold effects. The possible sources of nonthreshold effect heterogeneity included study design, methodologic study quality, and diagnostic criteria for PET timings. We used diagnostic odds ratio (DOR) to analyze the heterogeneity from nonthreshold effects. The Cochran-*Q* = 2.39 and *P* = 0.9348 (as shown in Figure 2), which indicated that there was no heterogeneity from nonthreshold effects.

**Figure 3:**
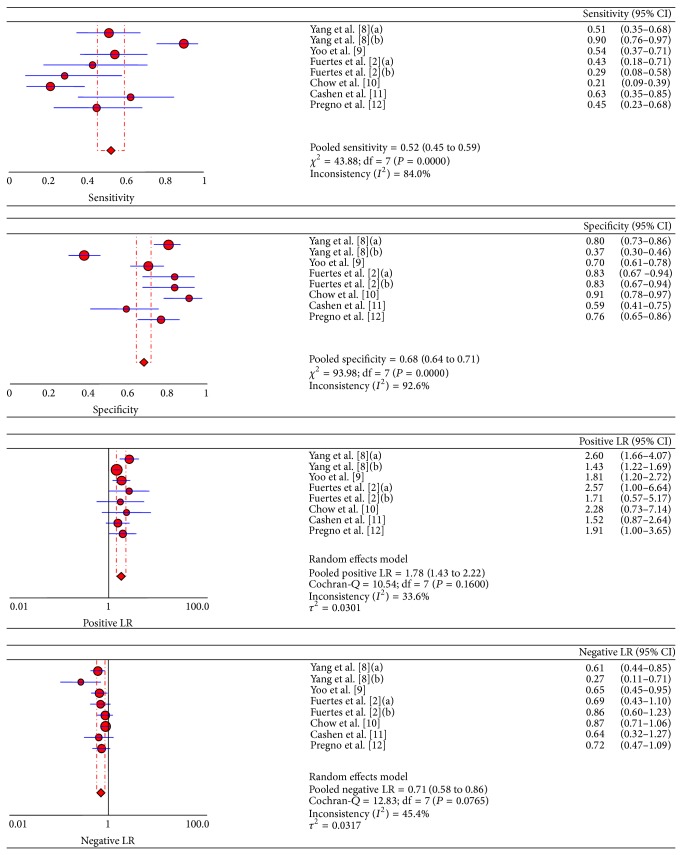
Sensitivity, specificity, and LRs of 6 included studies for assessing the prognostic value of interim PET/CT. Two studies [2, 8] used both visual and semiquantitative methods (ΔSUV_max⁡_) and the data of both methods was separately extractable. (a) is the visual group and (b) is the semiquantitative group.

We used a random effect model to calculate pooled sensitivity on the basis of statistical heterogeneity (*χ* ^2^ = 43.88, *P* < 0.05) and pooled specificity on the basis of statistical heterogeneity (*χ* ^2^ = 93.98, *P* < 0.05). As shown in Figure 3 and Table 2, the sensitivity of interim PET/CT ranged from 21.2% (95% CI, 9.0%–38.9%) to 89.7% (95% CI, 75.8%–97.1%) and the pooled sensitivity was 52.4% (95% CI, 45.4%–59.3%). The specificity of interim PET/CT ranged from 37.4% (95% CI, 29.6%–45.8%) to 90.7% (95% CI, 77.9%–97.4%), and the pooled specificity was 67.8% (95% CI, 64.0%–71.5%). The pooled positive likelihood ratio (LR+) and negative likelihood ratio (LR−) were 1.780 (95% CI, 1.427–2.221) and 0.706 (95% CI, 0.582–0.855), respectively.

**Figure 4:**
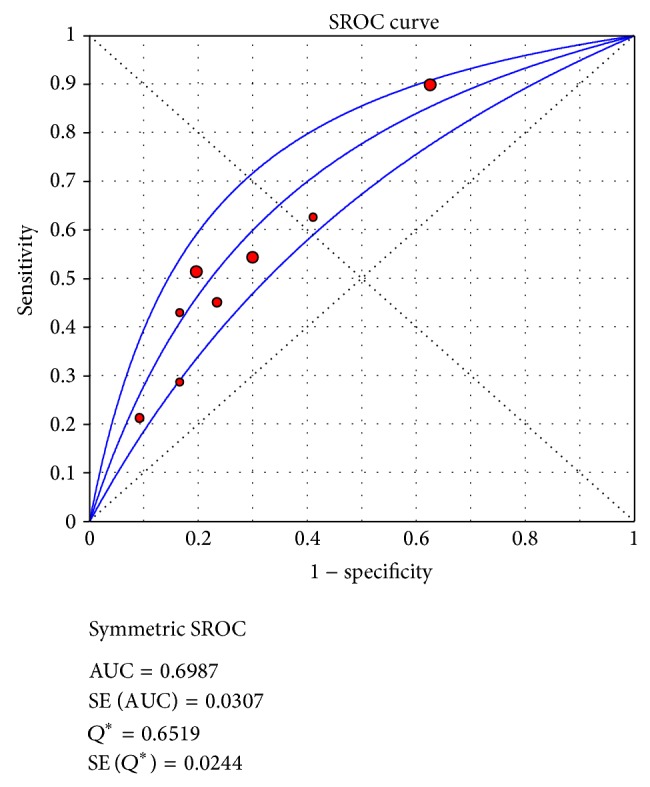
SROC of 6 included studies for assessing the prognostic value of interim PET/CT.

The AUC is used to summarize the overall diagnostic accuracy. As seen in Figure 4, of 6 included studies, the AUC was 0.6987 and the maximum joint sensitivity and specificity, *Q* ^*^, was 0.6519.

## 4. Discussion

The prognostic value of interim PET/CT performed during first-line therapy of patients with DLBCL is still unclear. Previous studies showed poor reproducibility and inconsistent accuracy and sensitivity of interim PET/CT due to different treatment modalities and response criteria. In an attempt to standardize interim PET/CT reporting criteria, the “First International Workshop on Interim PET in Lymphoma,” created in 2009, developed a consensus of response criteria for the interim PET. The response criteria were mainly based on visual and semiquantitative analysis. The visual response criteria used the Deauville five-point scale (5-PS): 1, no uptake; 2, uptake ≤ mediastinum; 3, uptake > mediastinum but ≤liver; 4, uptake moderately increased compared to the liver uptake at any site; and 5, markedly increased uptake compared to the liver at any site and new sites and/or new sites of disease. As seen in Table 1, of the 6 included studies, 2 used the Deauville five-point scale (5-PS). For semiquantitative analysis, since maximal standardized uptake value (SUV_max_) is the most commonly used semiquantitative method of PET analysis in oncology, assessment of the decrease in SUV_max⁡_ after a few cycles of chemotherapy compared with basal or pretreatment SUV expressed as a percentage (ΔSUV_max⁡_) can be useful in interim PET evaluation [18]. Spaepen et al. [19] reported the value of interim PET in predicting the outcome of DLBCL patients who had been treated with different chemotherapy regimens using delta-SUV-based criteria. Lin et al. [20] found a ΔSUV_max⁡_ of 65.7% to be the best cut-off level for differentiating patients with good or bad prognosis, with a very high degree of interobserver reproducibility. However, since the patients included in the 6 studies ranged from 2004 to 2010, not each of them was evaluated with the response criteria developed by the “First International Workshop on Interim PET in Lymphoma,” which contributed to the heterogeneity from the threshold effect.

In this study, we selected newly diagnosed DLBCL patients treated with R-CHOP. Our research showed that, due to different response criteria, studies had obvious threshold effect. SROC was used to summarize the overall test performance; and AUC was calculated to evaluate the indicator. The significance of AUC was that the AUC in the region of 0.97 or above is considered to have excellent accuracy, an AUC of 0.93 to 0.96 is very good; an AUC of 0.75 to 0.92 is good; and an AUC of less than 0.75 should be cautiously evaluated for the test may have obvious deficiencies in accuracy and is approaching the random test [21]. With these criteria, the results showed that interim PET/CT had deficiencies in accuracy in predicting the outcome of DLBCL patients treated with R-CHOP with an AUC of 0.6987.

There are several potential limitations to conducting a meta-analysis of diagnostic tests. First, many studies did partially include a relevant patient population meeting all the inclusion criteria. For these studies, we contacted the authors for relevant individual patient or subgroup data. Unfortunately, we got no responses and cannot get enough evidences to confirm the prognostic role of interim PET/CT. Second, due to the fact that patients included in the above studies ranged from 2004 year to 2010 year, only 3 of the 6 included studies used semiquantitative analysis. Lin et al. [20] found that SUV-based assessment of therapeutic response during first-line chemotherapy improved the prognostic value of early ^18^F-FDG PET compared with visual analysis in DLBCL. Casasnovas et al. [22] showed that SUV_max_ reduction improved early prognosis value of interim positron emission tomography scans in diffuse large B cell lymphoma. So maybe some more research should be done to assess the prognostic value of interim PET/CT with semiquantitative analysis. Third, studies included were retrospective and we suggest that larger prospective, high-quality, and multicenter studies should be conducted for DLBCL.

## 5. Conclusion

Just as shown in our study, the pooled sensitivity and specificity of interim PET/CT in predicting the outcome of DLBCL patients treated with R-CHOP chemotherapy were not satisfactory as expected. To improve this, some more work should be done to unify the response criteria and some more research to assess the prognostic value of interim PET/CT with semiquantitative analysis.

## Figures and Tables

**Table 1:** Characteristics and quality score of selected studies.

Author	Year	Follow-up	*N*	Age	Male	Criteria for outcome assessment	Interim PET (cycle)	Time frame	Quality score QUADAS
Yang et al. [8](a)	2013	2-year PFS	186	61 (17–83)	80	5-PS Visual	3/4	2004.8–2010.12	12
Yang et al. [8](b)	2013	2-year PFS	186	61 (17–83)	80	SUV_max⁡_	3/4	2004.8–2010.12	12
Yoo et al. [9]	2011	3-year PFS + OS	155	56 (16–85)	87	Visual	2/3/4	2004.3–2009.4	11
Fuertes et al. [2](a)	2013	5-year PFS + OS	50	55 (21–79)	28	5-PS Visual	2/3	2004.7–2007.5	12
Fuertes et al. [2](b)	2013	5-year PFS + OS	50	55 (21–79)	28	SUV_max⁡_	2/3	2004.7–2007.5	12
Chow et al. [10]	2013	2-year PFS + OS	76	61 (16–87)	45	Visual (IHP)	3/4	2005–2010	12
Cashen et al. [11]	2011	2-year PFS	50	58 (29–80)	Unknown	Visual (IHP)	2/3	2005.3–2008.5	11
Pregno et al. [12]	2012	2-year PFS + OS	88	35 (>60) 53 (<60)	41	Visual (IHP) + SUV_max⁡_	2/3/4	2004.4–2009.10	13

**Table 2:** Sensitivity, specificity, and LRs of selected studies.

Author	Sensitivity	95% Cl	Specificity	95% Cl	LR+	95% Cl	LR−	95% Cl
Yang et al. [8](a)	0.513	0.348–0.676	0.803	0.729–0.864	2.599	1.662–4.065	0.607	0.436–0.846
Yang et al. [8](b)	0.897	0.758–0.971	0.374	0.296–0.458	1.434	1.217–1.689	0.274	0.106–0.710
Yoo et al. [9]	0.543	0.366–0.712	0.700	0.610–0.780	1.810	1.202–2.723	0.653	0.447–0.955
Fuertes et al. [2](a)	0.429	0.177–0.711	0.833	0.672–0.936	2.571	0.996–6.638	0.686	0.426–1.104
Fuertes et al. [2](b)	0.286	0.084–0.581	0.833	0.672–0.936	1.714	0.568–5.172	0.857	0.597–1.231
Chow et al. [10]	0.212	0.090–0.389	0.907	0.779–0.974	2.280	0.728–7.142	0.869	0.710–1.062
Cashen et al. [11]	0.625	0.354–0.848	0.588	0.407–0.754	1.518	0.873–2.638	0.638	0.319–1.274
Pregno et al. [12]	0.450	0.231–0.685	0.766	0.646–0.859	1.913	1.002–3.652	0.719	0.474–1.092
